# Market access of orphan drugs and the role of multi-criteria decision making

**DOI:** 10.1186/1750-1172-7-S2-A26

**Published:** 2012-11-22

**Authors:** Steven Simoens

**Affiliations:** 1 Research Centre for Pharmaceutical Care and Pharmaco-economics, KU Leuven, Leuven, Belgium

## Background

A number of factors play a role in market access of orphan drugs, such as the extent to which an orphan drug meets a medical need; existence of alternative health technologies; disease prevalence; number of orphan drug indications; and added clinical benefit of the orphan drug. How can these factors be taken into account in market access decisions given that there is uncertainty over which factors matter and given that not all orphan drugs meet these criteria to the same degree [1]?

## Materials and methods

Multi-criteria decision analysis is a technique which enables decision makers to consider multiple criteria in market access decisions. This technique brings together an expert panel which identifies the relevant decision-making criteria and their relative importance. The panel then quantifies the extent to which an orphan drug attains each criterion. The scores of the orphan drug on the different criteria are weighted according to their relative importance and an overall score for the orphan drug is computed. Drugs are ranked according to their score and resources are allocated based on this ranking until the budget is exhausted.

## Results

To the best of the author’s knowledge, no multi-criteria decision analysis has in practice been carried out for orphan drugs. However, the following paragraphs illustrate this technique by proposing and justifying three criteria that could be considered in orphan drug market access decisions, i.e. disease prevalence, existence of alternative health technologies, and repurposing.

With respect to prevalence, an economic rationale suggests that prices of orphan drugs used for rare diseases with higher prevalence should be lower than prices of orphan drugs used for rare diseases with lower prevalence. Similarly, the price of orphan drugs should reflect the combined prevalence across its indications. The existence of alternative health technologies is a second criterion to consider in market access decisions in the light of the impact of competitive pressures on orphan drug pricing. Finally, orphan drugs which were originally developed for a common disease, but later repurposed for a rare disease are likely to have incurred lower costs of research and development and, therefore, should claim a lower price than an orphan drug which has been developed uniquely to treat a rare disease.

## Conclusions

The use of multi-criteria decision analysis would enhance objectivity and transparency of market access decisions for orphan drugs by taking into account societal preferences about orphan drugs for rare diseases.
